# Supplementary figures and images for: Temporal transcriptional response of Candida glabrata during macrophage infection reveals a multifaceted transcriptional regulator CgXbp1 important for macrophage response and fluconazole resistance
Source: eLife. 2024 Oct 2;13:e73832. doi: 10.7554/eLife.73832 (PMC11554308; doi:10.7554/eLife.73832)

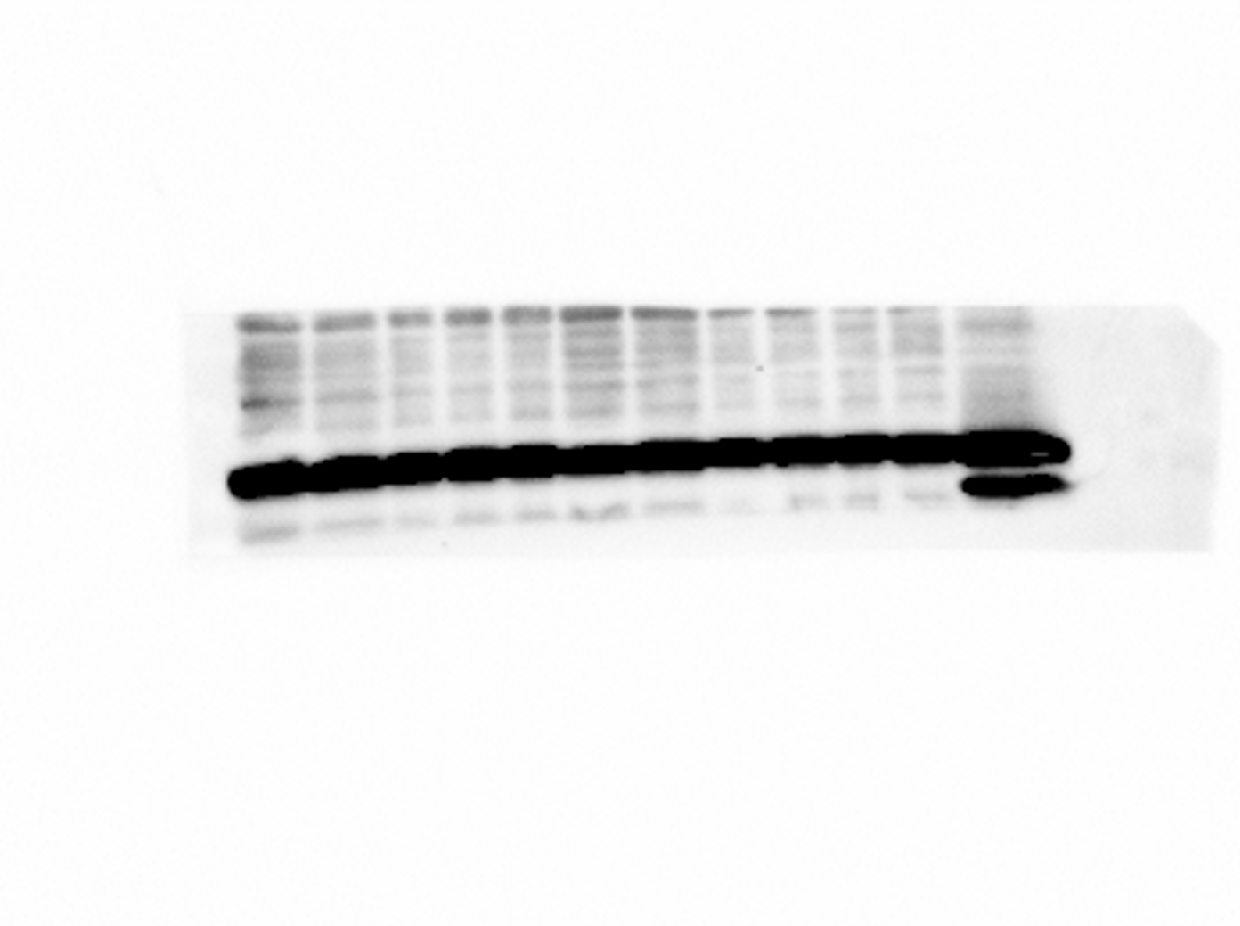

Supplement: Figure 2—source data 1. [file elife-73832-fig2-data1.zip › Figure 2-Source data 1/Figure 2-Source data_H3-control.tif]

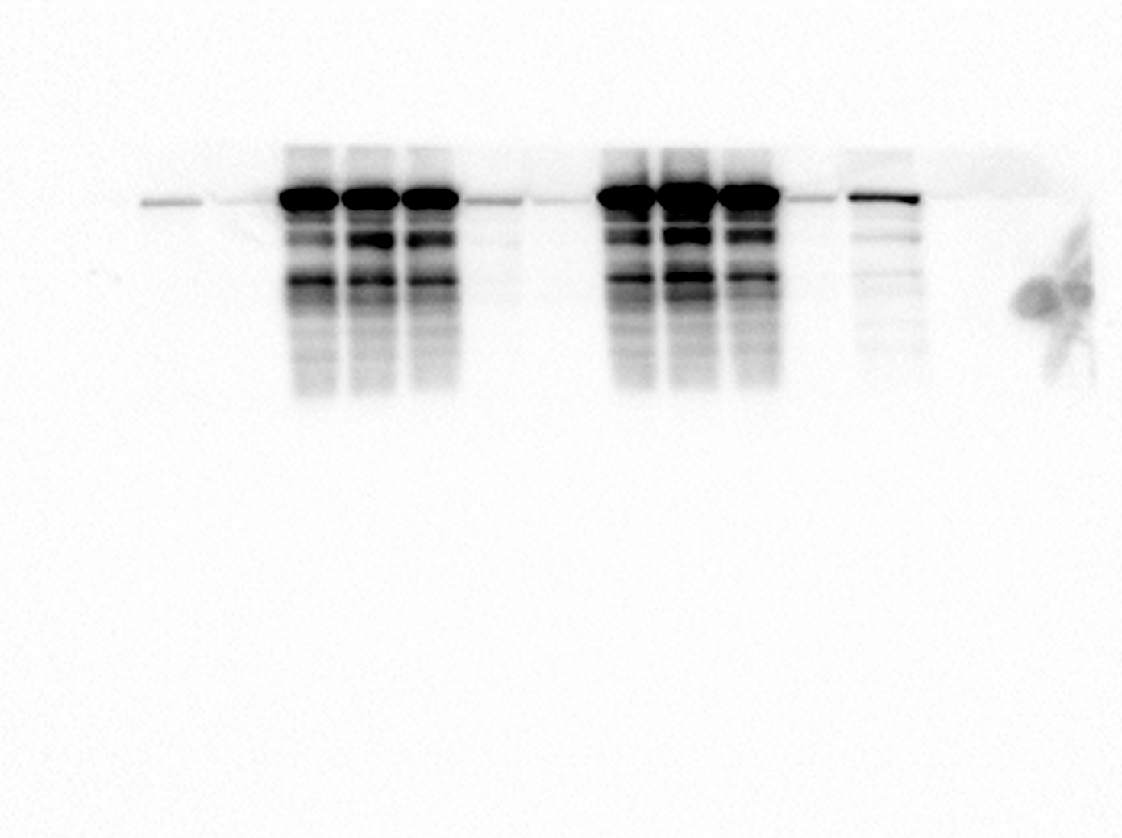

Supplement: Figure 2—source data 1. [file elife-73832-fig2-data1.zip › Figure 2-Source data 1/Figure 2-Source data_CgXbp1myc_western.tif]

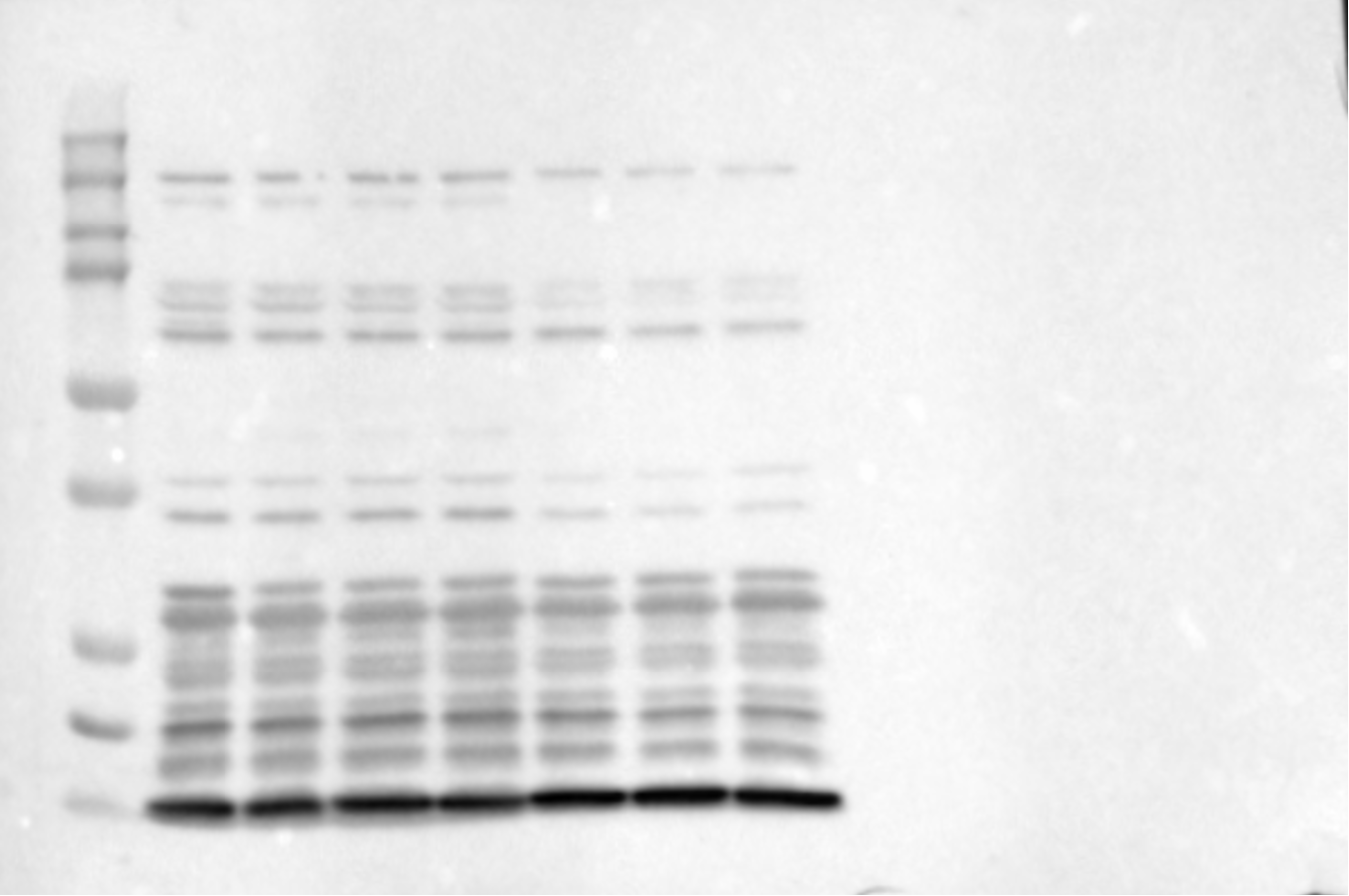

Supplement: Figure 6—source data 1. [file elife-73832-fig6-data1.zip › Figure 6-Source data 1/Figure 6-Source data_H3-control.tiff]

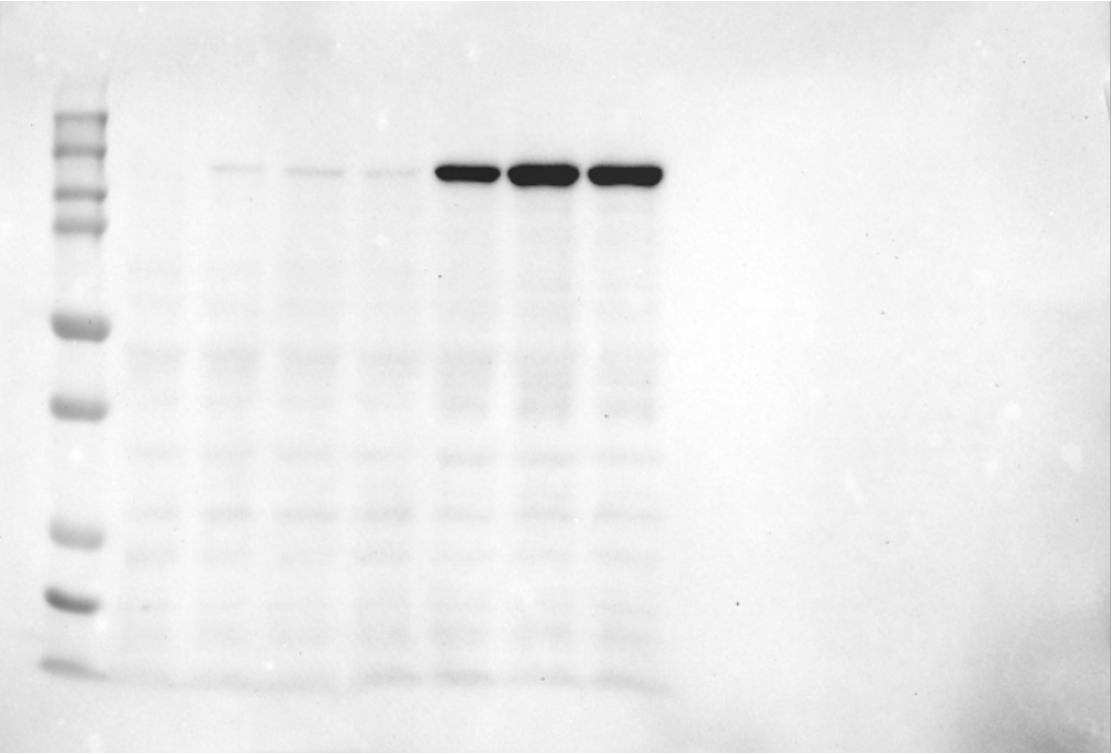

Supplement: Figure 6—source data 1. [file elife-73832-fig6-data1.zip › Figure 6-Source data 1/Figure 6-Source data_CgXbp1myc_western.tiff]
