# Supplementary material for: Temporal transcriptional response of Candida glabrata during macrophage infection reveals a multifaceted transcriptional regulator CgXbp1 important for macrophage response and fluconazole resistance
Source: eLife. 2024 Oct 2;13:e73832. doi: 10.7554/eLife.73832 (PMC11554308; doi:10.7554/eLife.73832)
Supplement: Figure 2—source data 2. [file elife-73832-fig2-data2.zip › Figure 2-Source data 2.docx]

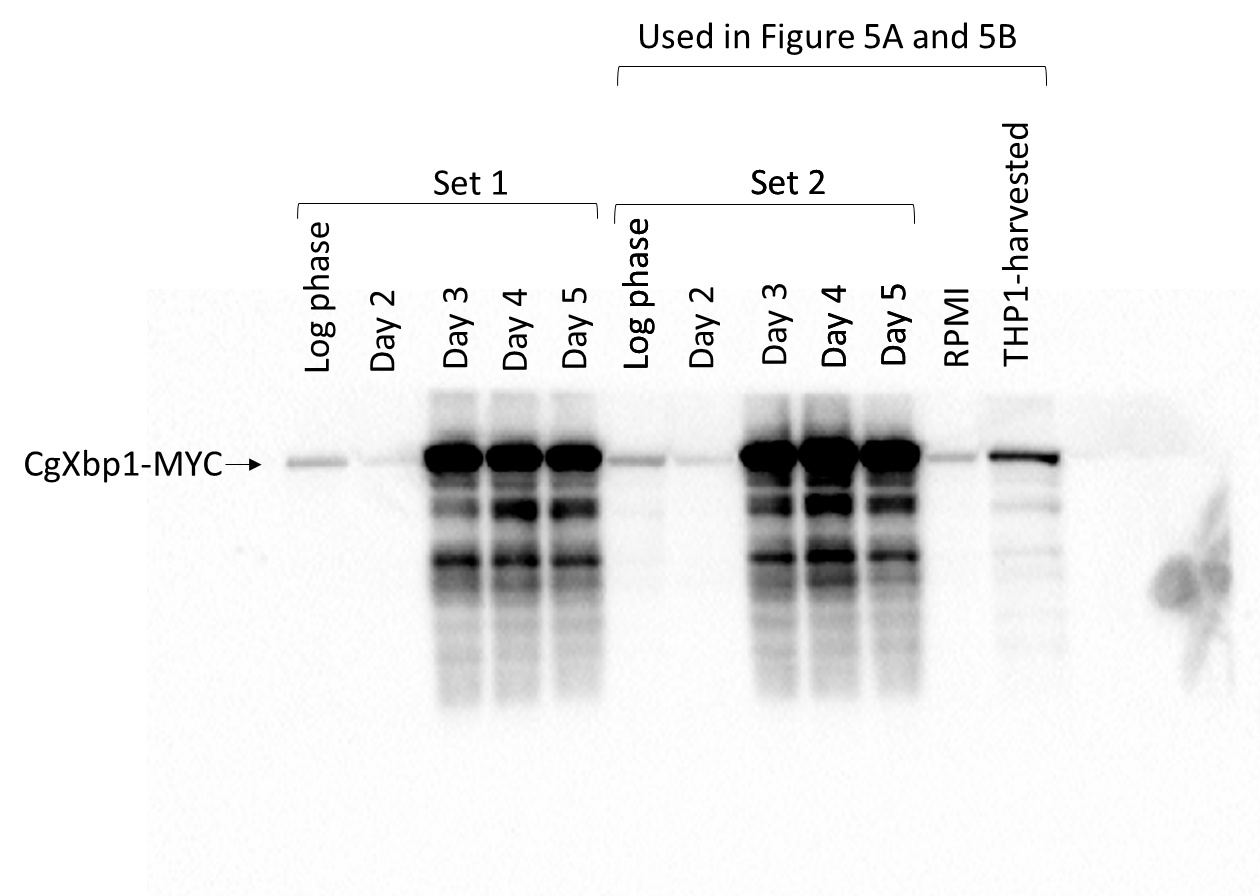


CgXbp1^MYC^ western, dotted box indicates lanes/samples used in Figure 2B.


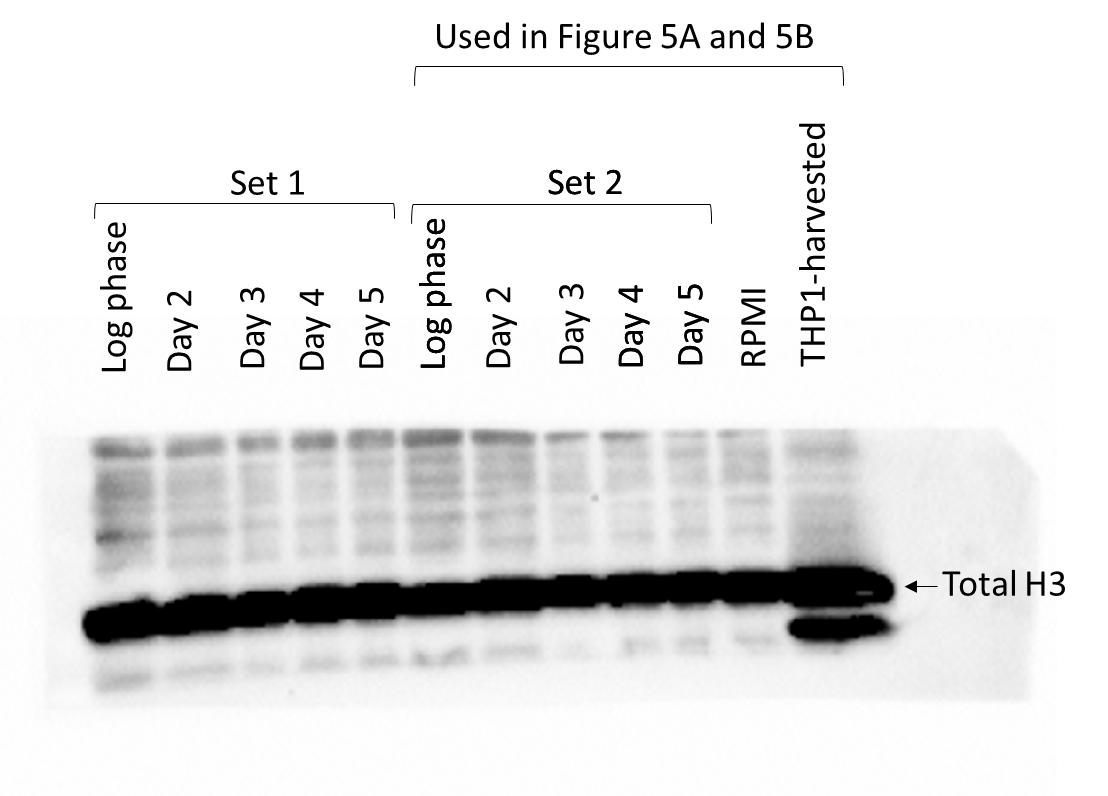


Histone H3 control, the dotted box indicates lanes/samples used in Figure 2B.
