# Supplementary material for: Temporal transcriptional response of Candida glabrata during macrophage infection reveals a multifaceted transcriptional regulator CgXbp1 important for macrophage response and fluconazole resistance
Source: eLife. 2024 Oct 2;13:e73832. doi: 10.7554/eLife.73832 (PMC11554308; doi:10.7554/eLife.73832)
Supplement: Figure 3—figure supplement 1—source data 1. [file elife-73832-fig3-figsupp1-data1.docx]

**Table (Panel B) in Figure 3-figure supplement 1**

| **GO-term** | **WT** | | | ***Cgxbp1∆*** | | |
| --- | --- | --- | --- | --- | --- | --- |
|  | **0.5h** | **2h** | **4h** | **0.5h** | **2h** | **4h** |
| small molecule metabolic process |  | Y | Y | Y | Y |  |
| generation of precursor metabolites and energy |  | Y | Y | Y |  |  |
| ATP metabolic process |  | Y | Y | Y |  |  |
| energy derivation by oxidation of organic compounds |  | Y | Y | Y |  |  |
| aerobic respiration |  | Y | Y | Y |  |  |
| ribonucleotide biosynthetic process |  | Y |  | Y | Y |  |
| purine-containing compound biosynthetic process |  | Y |  | Y | Y |  |
| ribose phosphate biosynthetic process |  | Y |  | Y | Y |  |
| purine nucleotide biosynthetic process |  | Y |  | Y | Y |  |
| oxidation-reduction process |  | Y | Y | Y |  |  |
| ATP synthesis coupled electron transport |  | Y |  | Y |  |  |
| lysine biosynthetic process |  | Y |  | Y |  |  |
| cellular amino acid biosynthetic process |  | Y |  | Y |  |  |
| ion transmembrane transport |  | Y |  | Y |  |  |
| small molecule biosynthetic process |  | Y |  | Y |  |  |
| alpha-amino acid biosynthetic process |  | Y |  | Y |  |  |
| carboxylic acid biosynthetic process |  | Y |  | Y |  |  |
| histidine biosynthetic process |  | Y |  | Y |  |  |
| transmembrane transport |  | Y |  | Y |  |  |
| aspartate family amino acid biosynthetic process |  | Y |  | Y |  |  |
| tricarboxylic acid cycle |  | Y |  | Y |  |  |
| acetate catabolic process |  | Y | Y | Y |  |  |
| nucleoside triphosphate biosynthetic process |  | Y | Y | Y |  |  |
| carbon utilization |  | Y | Y | Y |  |  |
| ribonucleoside triphosphate biosynthetic process |  | Y | Y | Y |  |  |
| trehalose biosynthetic process |  |  | Y | Y |  |  |
| response to oxidative stress |  |  | Y | Y |  |  |
| response to water |  |  | Y | Y |  |  |
| carbohydrate biosynthetic process |  |  | Y | Y |  |  |
| drug metabolic process |  | Y | Y |  |  |  |
| 'de novo' IMP biosynthetic process |  | Y |  | Y | Y |  |
| nucleoside phosphate biosynthetic process |  | Y |  | Y | Y |  |
| ribonucleoside monophosphate biosynthetic process |  | Y |  | Y | Y |  |
| fumarate metabolic process |  | Y |  | Y | Y |  |
| nucleotide biosynthetic process |  | Y |  | Y | Y |  |
| nucleosome assembly |  | Y | Y | Y | Y |  |
| monocarboxylic acid metabolic process |  | Y | Y |  |  |  |
