# Supplementary material for: Temporal transcriptional response of Candida glabrata during macrophage infection reveals a multifaceted transcriptional regulator CgXbp1 important for macrophage response and fluconazole resistance
Source: eLife. 2024 Oct 2;13:e73832. doi: 10.7554/eLife.73832 (PMC11554308; doi:10.7554/eLife.73832)
Supplement: Figure 6—source data 2. [file elife-73832-fig6-data2.zip › Figure 6-Source data 2.pdf]

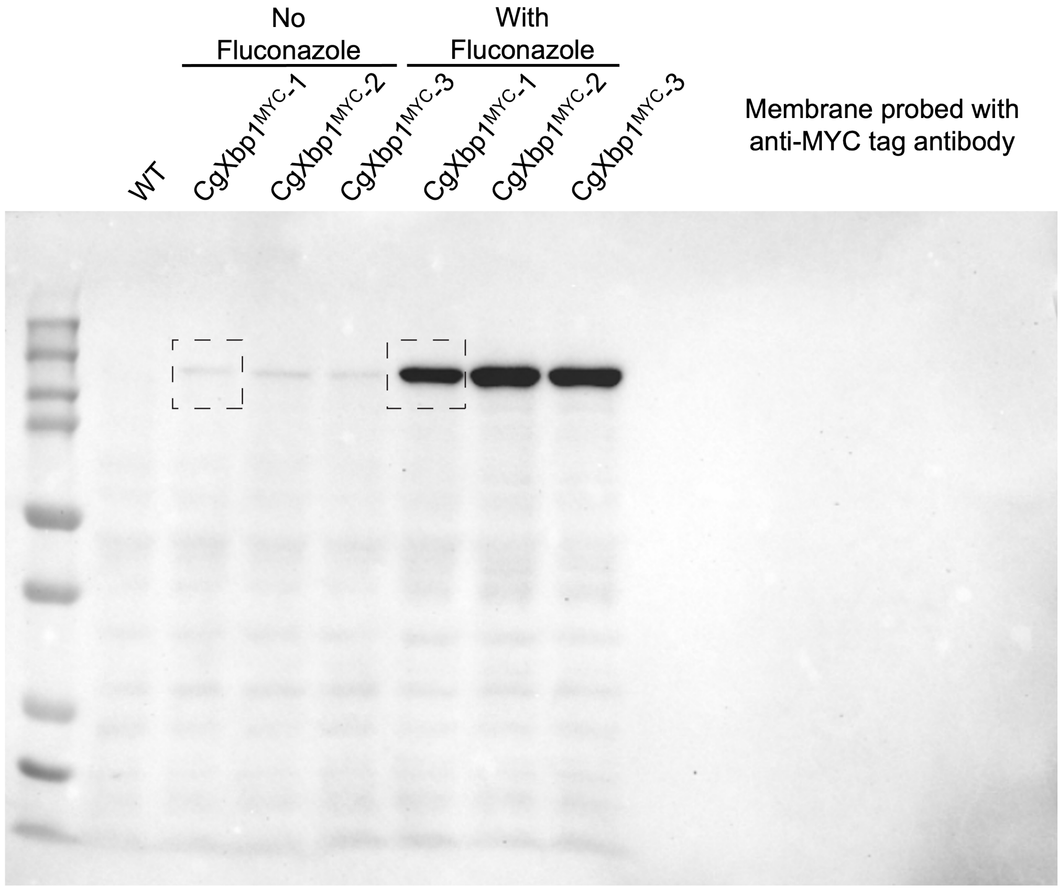

CgXbp1^{MYC} western, dotted box indicates lanes/samples used in Figure 6E.

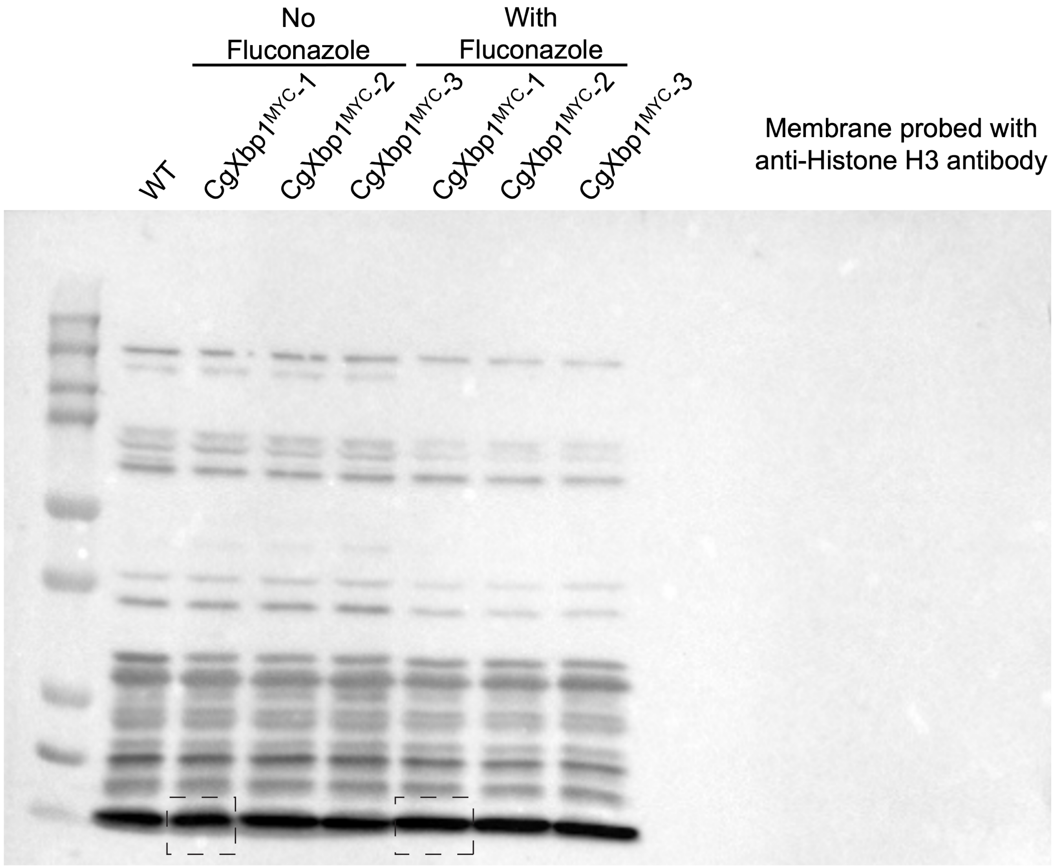

Histone H3 control, dotted box indicates lanes/samples used in Figure 6E.
